# Song preferences predict the quality of vocal learning in zebra finches

**DOI:** 10.1038/s41598-023-27708-y

**Published:** 2023-01-12

**Authors:** Carlos Antonio Rodríguez-Saltos, Aditya Bhise, Prasanna Karur, Ramsha Nabihah Khan, Sumin Lee, Gordon Ramsay, Donna L. Maney

**Affiliations:** 1 Department of Psychology, Emory University, 36 Eagle Row, Atlanta, GA 30322 USA; 2 Marcus Autism Center, Children’s Healthcare of Atlanta, Atlanta, GA 30329 USA; 3 Department of Pediatrics, Emory University, Atlanta, GA 30329 USA

**Keywords:** Animal behaviour, Learning and memory

## Abstract

In songbirds, learning to sing is a highly social process that likely involves social reward. Here, we tested the hypothesis that during song learning, the reward value of hearing a particular song predicts the degree to which that song will ultimately be learned. We measured the early song preferences of young male zebra finches (*Taeniopygia guttata*) in an operant key-pressing assay; each of two keys was associated with a higher likelihood of playing the song of the father or that of another familiar adult (“neighbor”). To minimize the effects of exposure on learning, we implemented a novel reinforcement schedule that allowed us to detect preferences while balancing exposure to each song. On average, the juveniles significantly preferred the father’s song early during song learning, before actual singing occurs in this species. When they reached adulthood, all the birds copied the father’s song. The accuracy with which the father’s song was imitated was positively correlated with the peak strength of the preference for the father’s song during the sensitive period of song learning. Our results show that preference for the song of a chosen tutor, in this case the father, predicted vocal learning during development.

## Introduction

Conspecific signals are attractive to receivers. Research on this attraction has focused mostly on signals such as courtship displays^1^ or foraging calls^2^. For both types of signals, attending to them may provide immediate benefits to the receiver. However, being attracted to conspecific signals is also important when the benefits are not immediate. Consider vocal learners, such as seals, bats, songbirds, and humans^3,4^. Juveniles of these species must attend to signals, such as speech or song, in order to imitate them; the benefits of doing so are often seen in adulthood, once these signals can be used to share information^2^, attract a mate, or secure a territory^5^. Thus, learning these signals likely depends on being attracted to them at a time when those benefits of signaling are beyond reach^6,7^. There should, therefore, be a great deal of selection pressure on young learners to be attracted to these signals, even in the absence of immediate mating opportunities or food rewards.

Songbirds lend themselves well to studying the processes by which attraction to song contributes to vocal learning. Zebra finches (*Taeniopygia guttata*), which are among the most commonly studied songbirds in the lab, are actively engaged in learning to sing. They memorize songs during social interactions with adults, and their degree of attention towards adult song “tutors” during these interactions predicts the quality of song imitation^8^. Juvenile finches are easily lured to press keys that elicit playback of song, and if given the opportunity, they will elicit playback hundreds of times per day. The fact that young finches are willing to work to elicit playback of song shows that this stimulus is rewarding to them, just as access to food or mates is rewarding to animals that are willing to press levers to obtain them. We hypothesize that the degree to which a particular song is rewarding predicts the degree to which it is learned.

Song learning is thought to rely on a mechanism similar to filial imprinting^9^ in that it is shaped by social experience during an early critical period. In mammals as well as in birds, imprinting is part of an overarching model of experience-dependent brain development in which early attachment, proximity-seeking, and social orienting contribute to learning^10–12^. Because song learning in zebra finches depends critically on such processes^6,9,13^, this species presents powerful opportunities to understand socially-guided vocal development. Beginning at around 20 days post-hatch (dph), before they are able to sing, young pupils select an adult male tutor and begin to memorize his song (only the males sing). The chosen tutor is nearly always the father. Once a juvenile has early experience with the father, if the father is removed before song memorization is complete, the juvenile will choose a tutor that looks like the father^14^ or sounds like him^15^. If a juvenile is reared from hatch by a foster male, he will learn the song of that male even if the biological father’s song is also heard in the room^16,17^. The influence of social interactions during learning is further evidenced by the fact that imitation is profoundly reduced when songs are presented via passive playback (e.g.,^8^); in some studies, finches that were passively tutored produced songs similar to those of finches that were not exposed to song at all^13,18^. Together, these studies show that the learning process is strongly shaped by the quality of social interactions, which may make one potential tutor’s song more rewarding to hear than another’s. In other words, early experience with the father is likely to lead to preferences for the father’s song, which could lead to enhanced learning of that song.

Preference for the song of the father, in the absence of the father himself, has been studied in finches both by measuring phonotactic responses^19–21^ and by using operant conditioning techniques^13,22,23^. In the Bengalese finch (*Lonchura striata domestica*), a species that is closely related to the zebra finch, juveniles more frequently approached speakers playing the father’s song than speakers playing unfamiliar songs^22^. This preference may result from the father’s song being more rewarding than other conspecific songs. It is still unclear, however, whether preference for rewarding songs contributes to the learning of those songs. Adret^13^ and Terpstra et al.^23^ used key-pressing assays to detect preferences in adult male zebra finches that had already finished learning to sing. These adults preferred the songs with which they were tutored over the songs of other adults^13,23^, but their preferences as adults did not predict the quality of learning^23^. Although these results are intriguing, they do not indicate whether preferences in adulthood reflect preferences during learning. Importantly, we do not know whether early preferences predict learning.

**Figure 1:**
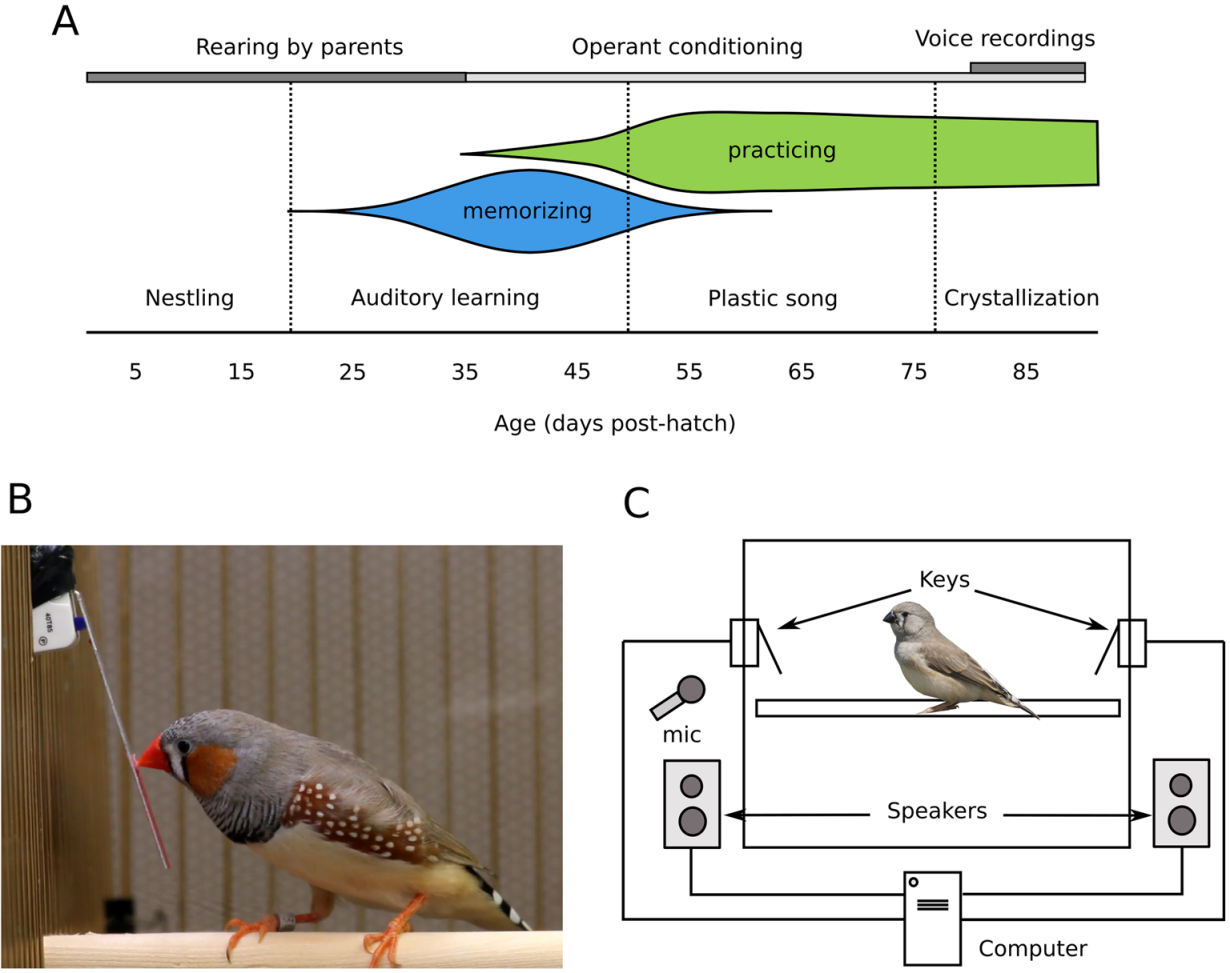
Experimental set-up. (**A**) Experimental timeline (top) and developmental trajectory of song learning in male zebra finches. Zebra finches were reared by their parents in a room where they could also listen to, but not see, adult male zebra finches other than the father (“neighbors”). At 35–37 dph, when finches are nutritionally independent^37^, the juveniles were transferred to an operant chamber equipped with keys that were associated with playback of father’s or neighbor’s song. Preference for father’s or neighbor’s song was measured daily while the bird remained in the operant chamber, until 90 dph. This timeline of operant conditioning covers most of the developmental trajectory of song learning. At the beginning of our assay, at 35–37 dph, zebra finches are still in an auditory phase of learning, during which they are known to memorize adult song^66^ and start practicing singing^37^. Singing rates start increasing by age 50 dph. Approximately at the same time, the finches enter the “plastic song” phase^26,27^, during which some memorization may still occur but ends by 65 dph^66^. Finally, song crystallization begins by 77 dph and finishes by 90 dph^26,37^. In the crystallization phase, between 80 and 90 dph, we recorded vocalizations of the juveniles and compared them to father’s and to neighbor’s song. (**B**) An adult zebra finch presses a key in an operant chamber to elicit playback of conspecific song. The same setup was used with the juveniles in our experiment. Photo by CAR-S. (**C**) The operant chamber consisted of a 14 × 15 × 17 inch cage, inside which two keys were placed on opposite walls. One key was associated with playback of father’s song and the other with playback of neighbor’s song. Outside the cage, one speaker assigned to each key played the songs. Photo of finch by Lip Kee Yap, shared under the Creative Commons Attribution-Share Alike 2.0 Generic license.

It may seem logical that birds learn their favorite songs best. This prediction is difficult to test, however, because in traditional preference assays, preference is confounded with exposure. If an animal chooses one stimulus over another, indicating preference, that animal exposes itself to the preferred stimulus more than the non-preferred one. Because exposure to a song^24^ and familiarity with it^25^ can affect learning, we developed a novel reinforcement schedule that allowed us to measure preference while balancing exposure. Using a key-pressing assay in juvenile male zebra finches, we tested preferences for the father’s song over that of a familiar, unrelated male. We measured preference each day beginning at nutritional independence, before juveniles of this species begin singing, and ending at song crystallization, when song is fully learned and does not change thereafter (Fig. 1A)^26–29^. At that point, we recorded the adult song to test whether early song preferences, shaped by high-quality social interactions, predict learning of that song.

## Results

### Developmental trajectories of song preference

**Figure 2:**
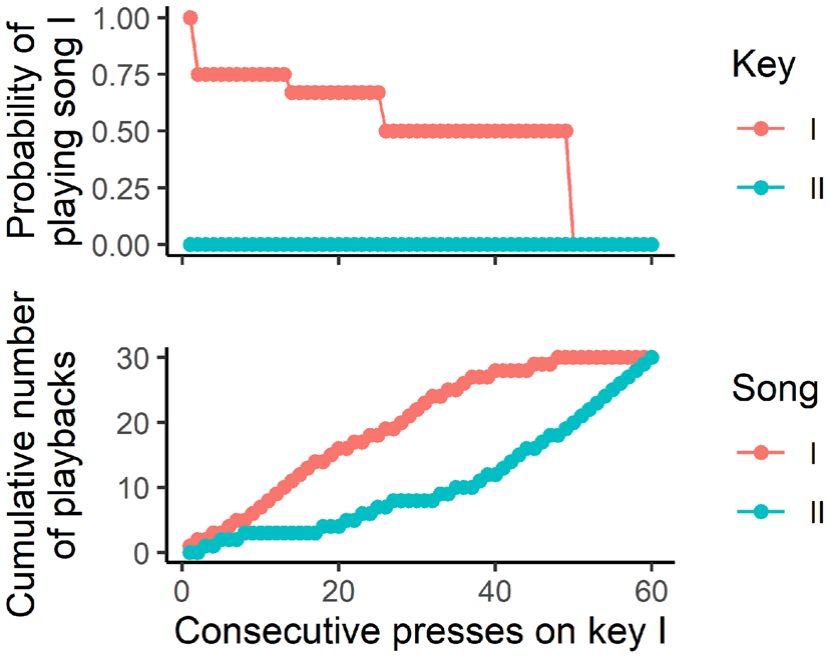
Reinforcement schedule to detect song preference while balancing exposure. A bird is presented with keys I and II, which are associated with a higher probability of playing song I and song II, respectively. Here we present an extreme example to illustrate how the schedule is able to balance exposure despite a strong preference. In this scenario, the bird prefers song I and presses key I only. The probability of playing song I by pressing that key is high at the beginning of the session, to help the bird form the association between that key and the song. As the bird keeps pressing key I, the probability decreases step-wise from 1 to 0.5, to prevent song II from lagging far behind in the playback count. This decrease, however, is not enough to balance exposure, and therefore, if the bird switches keys, key II plays only song II until the playback count of song II is balanced with song I. After enough presses on key I, song I eventually reaches a quota of 30 playbacks and ceases to be played. Afterwards, only song II is played, until that song also reaches the quota. Importantly, until the quota of the preferred song is exhausted, there is always a large difference between the keys with respect to the probability of hearing the preferred song. When the key associated with preferred song is playing that song only 50% of the time, the other key plays non-preferred song 100% of the time. See the Supplemental Methods for details about the reinforcement schedule. Key presses occurring after the quota of the preferred song was exhausted each day were not used in the analysis.

Juvenile male zebra finches, or “pupils”, were reared by their parents, with full access to the father, until nutritional independence (median age 37 dph, range: 35–37 dph). They were then transferred to operant cages containing two keys (Fig. 1B,C). One of the keys was more likely to play the song of the father (hereafter referred to as “father’s song”). The other key was more likely to play the song of a different male (hereafter referred to as “neighbor’s song”) that had been housed in the same room, but not the same cage, as the juvenile’s family cage and which had a song rate similar to the father. Each day, the pupil could press the keys to trigger 30 playbacks of each song. Because of our novel reinforcement schedule (Fig. 2; see Methods), exposure to each song was balanced each day. Each pupil heard 30 playbacks of father’s song and 30 of neighbor’s song, then the keys would trigger no further playbacks until lights-on the following day.

After being transferred to the operant cages the pupils engaged with the task and were exhausting the daily quota of 60 playbacks (30 of each song) within 5.0 ± 3.72 days (mean ± standard deviation). The age at which they started exhausting the quota did not correlate with the age at which they were transferred to the operant conditioning cages (Spearman’s rho = − 0.32; *p* = 0.31). Pupils remained with the operant conditioning task until a median age of 89.50 dph (IQR: 80.90–90.00). This duration of time spanned the three well-documented phases of song learning in this species: the auditory learning phase (in this case, the portion of that phase taking place after nutritional independence), the plastic song phase, and the song crystallization phase (in this case, the early portion thereof). These phases are described further in Fig. 1. One juvenile, which did not exhaust the quota of both songs after 13 days of being housed in the cage, was excluded from the study, leaving n = 12 that completed the key-pressing portion.

**Figure 3:**
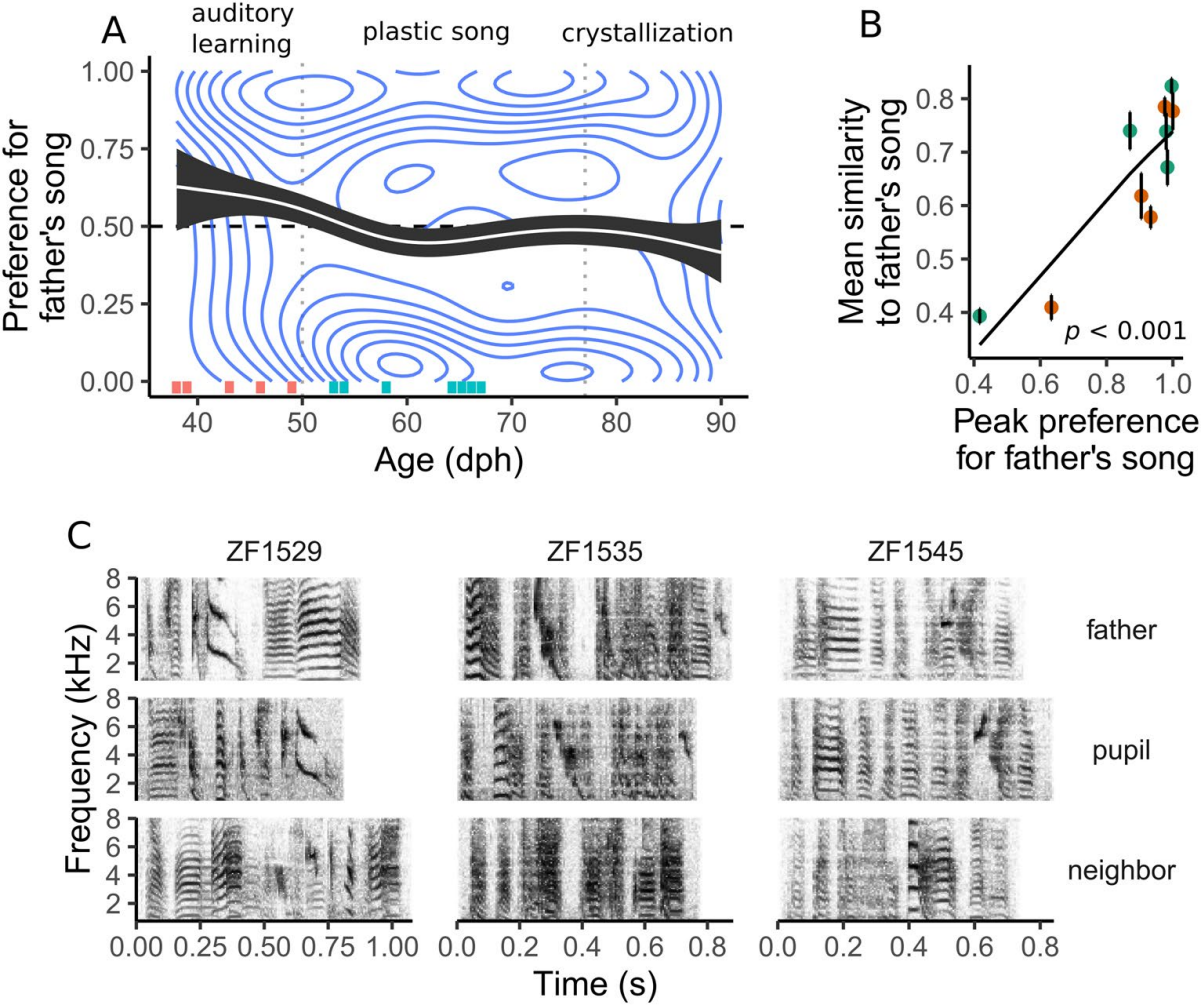
Relationship between the preference for father’s song and imitation of that song. (**A**) The white line shows the average trajectory of the preference for father’s song. The black area indicates the 95% confidence interval. The horizontal dashed line indicates no preference for either song. Vertical dashed lines indicate boundaries between developmental phases (see Fig. 1A). Concentric blue lines represent the 2D density kernel estimation of the daily preference scores. Ticks near the bottom of the plot indicate the age at which individual birds reached their peak preference for father’s song prior to the crystallization phase (for individual trajectories, see Fig. S2). Given that crystallization may begin as early as 70 dph^26^, we chose the maximum preference reached before that age (see Fig. S5 for an alternative analysis using the peak preference over the entire learning trajectory, including crystallization). Ticks are drawn with an offset to prevent overlap. Preferences peaking during the phase known as auditory learning (see Fig. 1) are marked in orange, and those peaking during the plastic song phase are marked in teal. (**B**) Peak preference for father’s song was significantly correlated with mean similarity to that song. The curve is the fit of a beta regression. Vertical bars show the standard error of the mean similarity score. Data points are colored according to the phase during which the bird reached maximum preference for father’s song, as indicated in (**A**). (**C**) Spectrograms of the songs of three pupil exemplars are shown next to the spectrograms of the songs of their respective father and neighbor. The spectrograms indicate that these three pupils imitated father song and not neighbor song. All other pupils also imitated father song or, in the case of one individual, did so most of the time (see text). Spectrograms were generated in R^73^ using Hanning windows containing 512 samples each (11.61 ms for recordings sampled at 44,100 kHz), with 50% overlap between windows.

On the basis of previous research^19,20,30^ we hypothesized that the juveniles would show a preference for father’s song over neighbor’s song, and we therefore calculated preference with respect to father’s song (preference for neighbor’s song is simply the preference for father’s song subtracted from 1). For the 12 pupils that reliably exhausted the quota of both songs each day, we estimated the strength of the preference for father’s song over neighbor’s song daily by calculating the proportion of presses on the key associated with father’s song (Fig. S1). Presses occurring after the quota of the preferred song was reached were excluded from this calculation. Scores above 0.5 indicate that father’s song was preferred over neighbor’s song. On average, the father’s song was preferred over neighbor’s song between ages 40–50 dph (preference for father’s song > 0.5; 0.5 outside 95% confidence interval [CI]) (Fig. 3A), during the phase of learning known as the auditory phase^26^. The neighbor’s song was preferred between developmental ages 60–65 dph (preference for father’s song < 0.5; 0.5 outside CI). The shift from preferring father’s song to then preferring neighbor’s song was also seen in many individual developmental trajectories (Fig. S2), although the timing of this developmental milestone varied from bird to bird as is common for developmental processes^31^.

### Correlation between preference and imitation

We next tested whether the strength of the preference for a song predicted the quality of imitation of that song. As a measure of preference, we identified the maximum value on a smoothed trajectory of preference for father’s song for each pupil. The maximum peak preference occurred during the auditory phase of learning for five pupils, during the plastic song phase for five pupils, and during crystallization for only two (see Fig. 1 for definitions of the phases; see Fig. S2 for individual trajectories of preference). During crystallization and the days leading up to it, learning ends and the song becomes fixed^32–34^. The crystallization phase begins between 70 and 77 dph^26^. Therefore, we did not consider peak preferences after 70 dph relevant to learning. For pupils with peak preferences after 70 dph (Fig. S2), we instead used their peak preference values before 70 dph (each peaked during plastic song) in this analysis.

For most of the pupils, the maximum preference was above 0.91, reflecting a strong preference for father’s song. Only one pupil had a maximum preference below 0.5 (Fig. S2), meaning that it always preferred neighbor’s song over father’s song. Maximum preference for father’s song was not correlated with the age at which it was reached (Spearman's rho: − 0.01, *p* = 0.97).

We next quantified imitation by examining the similarity^35^ between each pupil’s song and the corresponding two tutors’ songs (father’s and neighbor’s). To determine which tutor’s song was more similar to the pupil’s song, we used Sound Analysis Pro 2011 (SAP2011)^36^ to compare each juvenile’s crystallized song with the recordings of its father and neighbor that were used in the preference assays. The pupils’ songs were, in each case, more similar to father’s than to neighbor’s song (*p* < 0.001). The mean similarity score, per bird, was 65.3 ± 1.5 (mean ± s.d.) for father’s song and 56.6 ± 18.1 for neighbor’s song. The similarity was also supported by visual comparisons of spectrograms of pupil’s, father’s, and neighbor’s songs (Fig. 3C, S3). All pupils shared syllables in their songs with father’s song. The song of just one pupil contained a single element, that is, part of a syllable, from neighbor’s song (Fig. S4). Thus, we are confident that pupil’s songs in our study more closely resembled father’s song than neighbor’s song.

To test whether the strength of the preference for the tutor’s song predicted the quality of imitation of that song, we fitted beta regression models to our data on maximum preference and average similarity, controlling for brood as a random effect. Because technical issues prevented us from recording the crystallized songs of two pupils, our final sample size for this analysis was n = 10. Despite this reduction in sample size, our analysis for father’s song achieved power of 0.96. We found a large, significant correlation between maximum preference for father’s song and average similarity of pupil’s song to father’s song (adjusted R^2^ = 0.73; *p* < 0.001, Fig. 3B). One data point was found to be an outlier influencer in the correlation (Cook’s distance > 3 * average Cook’s distance), but even after removing that point, the correlation remained significant. In contrast, we did not find a significant correlation between maximum preference for neighbor’s song and average similarity of pupil’s song to neighbor’s song (adjusted R^2^ = − 0.125; *p* = 0.90; power = 0.05). The correlation between maximum preference for father's song and average similarity to father's song remained significant when including peak preferences after 70 dph, during the song crystallization phase (Fig. S5).

### Relative importance of live tutoring vs. operant tutoring

Two birds not included in this study were tested during operant conditioning using two neighbor songs. We compared their crystallized songs to that of their father and the two neighbors. Despite not having had exposure to their father’s song after 35dph, and hearing only neighbor’s song during operant conditioning, the songs of these juveniles were similar to father’s song (Fig. S6). We did not find elements from the two neighbor’s songs in the pupils’ songs.

### Discussion

#### Preferences and vocal learning

In this study, we showed that juvenile male zebra finches, when socially reared with the father until 35 dph, preferred to hear father’s song while in the auditory phase of song learning (Fig. 1A) and ultimately sang father’s song. Our finding that the pupils preferred father’s song is consistent with other studies using a variety of methods of assaying preference^13,20^, and in particular with a recent study showing that the preference for tutor song may peak early during vocal development^22^. Our result that all of the pupils imitated the father is consistent with other reports suggesting that the quality of early social experiences, which is presumably higher for interactions with the father than with other males, is critically important for tutor choice and high-quality song learning^14,15,37–39^.

Our most important finding is that the peak preference for father’s song strongly predicted learning of that song (Fig. 3B). Our interpretation of this finding is that the incentive salience of a song stimulus, in other words the pupil’s willingness to perform work to hear it, may facilitate vocal learning. We have previously hypothesized that vocal learning in songbirds is facilitated by the naturally rewarding properties of conspecific song, particularly the song of a caregiver during early development^40,41^. In this study, the development of song preference was pupil-driven, not driven by exposure. Because exposure to father’s versus neighbor’s song was balanced in our paradigm, we conclude that the decisions about what was most attractive and what to learn were made by the pupil, based on the pupil’s social history with each singer. Unlike preference assays that use perch selection or proximity to indicate preference^42^, in our assay, the birds could choose not to hear song at all. Because all of the birds chose to key-press, we believe their preferences for a particular key resulted from positive, not negative, reinforcement. We do not know whether a preference for father’s song represented an attachment to the father himself or simply a desire to hear the song that the pupil wished to learn. In fact, because positive feedback from the mother to the father may be perceived by the pupil^43,44^, we cannot say whether the father himself played any role in the development of preference for his song. Regardless of the mechanism, however, it is clear from our results that the strength of the juvenile’s preference predicted the quality of learning.

These results may have interesting implications for the development of speech in humans. Like song learning in songbirds, speech learning in humans depends critically on early social interactions. It has been hypothesized that the motivation to attend to social stimuli plays an important role in learning speech. For example, gaze following, the desire to imitate a caregiver, and joint attention between a child and a caregiver predict language acquisition^45^. Alterations in social orienting, which likely indicate alterations in social reward, are thought to cause children with autism to develop speech more slowly relative to typically developing children^46,47^. Thus, zebra finches may serve as important models for the role of social reward in vocal learning in humans.

### Developmental trajectory of song preferences

In zebra finches, auditory learning takes place roughly between 25 and 60 dph (Fig. 1). During this phase, pupils begin to perform subsong, an early form of singing characterized by high variability and lack of distinguishable phrases^37^. The critical process going on during this phase is likely not vocalizing, but listening; previous work in this species has shown that pupils engage in the most effective song memorization at around 35–45 dph^48^, a time that, in our study, corresponded with a clear peak in preference for father’s song (Fig. 3A). Thus, the peak in the incentive salience of that song may happen at around the same time as the known peak in memorization. We noted that for some pupils, preference for father’s song peaked immediately after learning the operant task (Fig. S2). For those birds we assume that preference for father’s song was high even before they entered the assay, consistent with the auditory learning phase beginning before independence from the parents^49^.

Beginning around 50 dph, we noted a change in the average song preference. At that time the average preference moved toward neighbor’s song and was statistically significant by 60 dph. This developmental time point is an important one in many respects. Other studies have shown that it coincides with the onset of “plastic song”, a phase in which the juvenile’s song rate increases dramatically^26,37^ and syllable structure begins to resemble that of the tutor^27^. Although we did not analyze the juveniles’ vocalizations during this period, our results suggest that during this period, pupils are spending less time seeking out the song they will eventually sing. Perhaps their efforts have shifted toward practicing rather than listening. The shift in preference, toward neighbor’s song, is interesting because on average it occurred at a time when juvenile males typically transition from spending time with the family unit to seeking contact with other, unrelated birds^50^. This transition may be reflected in their song preferences; Fujii et al.^22^ showed that preferences for the father’s song over that of an unfamiliar male began to wane after 60 dph in males but not in females.

### Potential neural mechanisms

Our findings are consistent with a model in which early social interactions with tutors increase the incentive salience of tutor song, which in turn strengthens the formation of auditory memories to facilitate learning. Several groups, including ours, have reported findings suggesting potential neural substrates. The memories for tutor song that guide vocal imitation are thought to reside in the auditory forebrain^23,51^, a region rich in neuromodulators known to mediate social reward. Our own work with adult white-throated sparrows has shown that catecholaminergic activity increases in the auditory forebrain in females during sexual receptivity, when hearing song is likely rewarding^52,53^ and that hearing song induces this activity further^54,55^. In young zebra finches, song learning may depend on catecholaminergic circuits; Katic et al. found that activity in the locus coeruleus, the source of noradrenergic input to the auditory forebrain, increases during live song tutoring and blocking presynaptic signaling in the auditory forebrain interferes with song learning^56^. Further, Phan et al. showed that the degree to which song-induced activity in the auditory forebrain is tuned to tutor song predicts the quality of song learning^25^; that is, the stronger the memories for tutor song, the better that song is learned—which echoes the findings we report here.

The strength of song memories may depend not only on catecholaminergic activity but also on the nonapeptide systems implicated in social reward across vertebrates. In a previous study^57^, we mapped and quantified the distribution of oxytocin receptor (OTR) expression during the entire period of vocal development in zebra finches. This receptor is highly expressed, early in development, in many brain regions important for song learning, including song control nuclei and the auditory forebrain. Interestingly, we found a striking reduction in OTR mRNA at ~ 55 dph in each of these regions. This result, together with our current finding of a shift in song preferences at the same age, provides correlational evidence that early attraction to father song is associated with OTR expression in some or all of these regions. Given the well-known role of OTR in social attachment and sociality^58^, which has been shown even in zebra finches^59,60^, it is possible that this receptor contributes to socially-mediated vocal learning by mediating the establishment of early song preferences^43,61^.

### Potential genetic contributions

A rich literature dating back several decades has shown clearly that tutor choice depends critically on the quality of early social interactions with adult males^14,15,37–39^. It is thought that young pupils choose to imitate their father because they interact with him the most. Young zebra finches choose to imitate the song of a foster father even if the biological father can be heard singing in the same room^17,62^. Nonetheless, there is recent evidence that certain components of song may have a genetic basis^63,64^. Because we did not cross-foster the pupils in our study to unrelated parents, we do not know the extent to which the preferences exhibited during the key-pressing assay could be explained instead by a genetic component. Future studies of song preferences should include cross-fostering to evaluate the extent to which experience contributes to the correlation between preference and learning that we report in this study.

### An operant conditioning assay to measure preference while balancing exposure

As part of this project, we developed an operant assay that can be used to measure preference for a stimulus while controlling for exposure to that stimulus. This assay may be useful to other researchers, not only those studying song learning but also any process in which the outcome measure may be confounded by exposure effects.

We note that in the present study, we may not have needed to control for exposure effects on learning. Two birds, which were excluded from our main analysis, were reared with the father and then given a choice between the songs of two neighbors in the operant assay. Both ultimately sang father’s song instead of either neighbor’s song. Although there were only two birds in this condition, this result adds an interesting layer to our knowledge about what can and cannot be accomplished by operant tutoring. We know from previous work that if the father is removed at 35 dph and a new, live tutor is provided, pupils will learn primarily the new tutor’s song^65,66^. Further, Varkevisser et al. recently showed that although song crystallization can be delayed if the pupil is visually isolated from the new tutor, ultimately these authors detected no decrement in the quality of learning compared with pupils with full audio and visual access^67^. In our study, the father was removed and replaced with only keys to press for novel playbacks, not live tutors. We found that under these conditions, these two pupils rejected the novels songs, choosing instead to sing father’s song. We see many possibilities for testing a variety of hypotheses with this assay and hope that others can use it in their own studies of preference.

## Materials and methods

### Ethics statement

All of our procedures involving handling and experimentation with animals were approved by the Institutional Animal Care and Use Committee at Emory University under protocol 2003144–052615 N. All methods were performed in accordance with the relevant guidelines and regulations. Reporting follows the recommendations in the ARRIVE guidelines^68^.

#### Animal housing

Adult breeding pairs were housed in 14 × 15 × 17 inch cages. All items in the cage, such as food trays, water baths, and bottles, were arranged symmetrically to discourage preferences for either side of the cage. Within a single room, four breeding pairs were housed together, each pair in its own cage. White plastic dividers placed between the cages prevented the birds from seeing each other, although they could hear each other. The four adult males housed inside any particular breeding room were intentionally chosen on the basis of dissimilar songs that we could easily distinguish from each other. Juvenile zebra finches can discriminate acoustic structure at a higher temporal resolution than can humans^69,70^; we thus expected that the juveniles in our study had no difficulty discriminating songs that we perceived as dissimilar.

### Operant chamber

We used only male pupils for this experiment because in this species, only males sing. Juvenile males (n = 13) were separated from their parents at 37 (IQR: 36, 37) days post-hatch (dph) (Fig. 1A). By this age, juvenile zebra finches can feed themselves^17^. Each male was isolated from other birds in a 14 × 15 × 17 inch cage placed inside a sound-attenuating booth. Isolation was necessary to prevent the juvenile from hearing other birds’ responses to song playback, which could influence the development of preferences. Food, water and other items in the cage were arranged symmetrically to reduce side bias. To provide enrichment, a mirror was centered on the rear wall of the cage.

The cage was equipped with two keys (Fig. 1B,C), placed on opposite walls. Upon being pressed, each key elicited playback of either the father’s song or the song of a neighbor with a similar song rate (see Supplemental Methods). One of the keys had a higher likelihood of eliciting playback of father’s song while the other key had a higher likelihood of eliciting playback of neighbor’s song. Whether the left or right key was associated with father’s or neighbor’s song was balanced across subjects. Acquisition of the playback stimuli triggered by key presses is described in the Supplemental Methods.

The keys were connected to a computer via a National Instruments Board USB-6501 (National Instruments, Austin, TX, USA) or an Arduino UNO board (Arduino LLC, Somerville, MA, USA). The computer ran the software SingSparrow!, which we wrote, to control the responses of the keys and to log the presses on them (code available in Supplemental Materials). Each cage had two speakers (LS-300, AudioSource, Portland, OR, USA; or Logitech Z200, Newark, NJ, USA), each one paired with one key. In each cage, the two speakers were of the same model. Operant conditioning proceeded daily until the bird reached 89 dph (IQR: 87, 90) (Fig. 1A), when song is crystallizing, or taking its final form^37^. Thus, operant conditioning proceeded throughout the majority of the period when the zebra finches practiced singing.

### Reinforcement schedule

To test whether preference predicts learning, we needed to control the amount of exposure to each song. Otherwise, a correlation between learning and preference may result from increased exposure to the preferred song. Because the father and the neighbor had similar song rates (see Supplemental Methods) the juveniles’ exposure to father’s and neighbor’s song was roughly equal while the juvenile lived in the breeding room. To control exposure during the operant conditioning phase of the study, we designed a reinforcement schedule that allowed us to detect a preference for either song, balance exposure to each song, and limit exposure to 30 playbacks of each song per day (Fig. 2). This quota of playbacks was chosen to prevent detrimental effects of overexposure on learning^24^. Each key in the operant conditioning cage could play both songs, but each had a higher probability of playing either the father’s or neighbor’s song. The probabilistic schedule allowed the birds to play both songs throughout the session while still indicating their preference for one of the songs. Once the quota of their preferred song was reached, the pupils could play only the other song by pressing either key, until its quota was also reached. Once the quotas of both songs were reached, pupils could not elicit any more playbacks that day. The keys were reset the following morning at lights-on.

### Reconstructing developmental trajectories of song preference

Logs of key presses were cleaned in three steps before they were used to estimate trajectories of song preference. First, we deleted days in which the pupils had not exhausted the quota of both songs. These days occurred mostly at the beginning of the experiment, while the birds were habituating to the cage and learning the operant task. Second, we removed presses that occurred after the quota of the preferred song for that day was exhausted, because at that point the pupils no longer had a choice between the two songs. Third, we removed presses that occurred within three seconds of a previous press because these presses were unlikely to be independent of the first. We had programmed the keys not to play more than one song within three seconds of a press, so these extra presses did not elicit playback.

To measure preference for either song, we calculated the daily proportion of presses on the key associated with that song. For pupils with strong preferences, we ruled out side biases as described in the Supplemental Methods.

To reconstruct the average trajectory of preference for father’s song, we fitted a generalized additive model (GAM) to our data^71^. The dependent variable was preference for the father’s song and the independent variable was age. Bird identity was modelled as a random effect. To constrain predicted values to the interval 0–1, we used the GAM beta regression family. The relationship between preference and age was modelled using a thin-plate regression spline^72^. The model was fitted using the library mgcv^73^ in R^74^. Mgcv produced a 95% confidence interval of the trajectory by multiplying the standard error of the trajectory by two, subtracting this result from the trajectory to find the lower bound, and adding it to the trajectory for upper bound^73^. At any given age the pupils significantly preferred the father’s song over the neighbor’s song when the trajectory was above 0.5 and the confidence interval excluded that value.

In order to find the maximum preference of each pupil for the song of the tutor it chose, we first fitted individual trajectories of preference for that song. For each bird, we fitted the trajectories using locally estimated scatterplot smoothing (LOESS)^75^ in R^74^ (Fig. S1). Smoothing the preference curves allowed us to increase the reliability of our estimates of maximum preference while at the same time preserving information about the developmental trajectory for each bird. The degree of smoothing was controlled via the span parameter, which was set using leave-one-out cross-validation. For each bird, we recorded the global maximum point of the trajectory and the age associated with it.

### Analysis of acoustic similarity and correlations with preference

Recordings of the vocalizations of pupils were made, using a microphone in front of the pupil’s home cage, between 80 and 90 dph (see Supplemental Methods). By 80 dph, song crystallization is well underway and the song is a reliable proxy for adult song^26,29,37^. We used SAP2011 to evaluate acoustic similarity between the songs of tutors and pupils^35,36^ as described in the Supplemental Methods. We then tested whether maximum preference for each tutor’s song was correlated with the pupil’s average similarity to that tutor’s song. The correlation was tested using a generative additive model with the package mgcv^73^ in R^74^. The model included a beta regression link and controlled for the random effect of brood. The power of the correlation was assessed using G*Power 3.1.9.7^76^. In that software, we chose the statistical test for linear multiple regression: Fixed model, R^2^ deviation from zero.

## Supplementary Information

Supplementary Information 1.

Supplementary Information 2.

## Data Availability

The datasets used and/or analyzed during the current study are available from the corresponding author on reasonable request.
