# Supplementary material for: Song preferences predict the quality of vocal learning in zebra finches
Source: Sci Rep. 2023 Jan 12;13:605. doi: 10.1038/s41598-023-27708-y (PMC9837092; doi:10.1038/s41598-023-27708-y)
Supplement: Supplementary file 1 — Supplementary Information 1. [file 41598_2023_27708_MOESM1_ESM.pdf]

Song preferences predict the quality of vocal learning in zebra finches

Carlos Antonio Rodríguez-Saltos, Aditya Bhise, Prasanna Karur, Ramsha Nabihah Khan, Sumin Lee, Gordon Ramsay, and Donna L. Maney

SUPPLEMENTARY METHODS

Playback stimuli

To identify the neighbor with the song rate closest to the father's, we estimated the singing rates of each of the four adult males in the room during the time that the juvenile was housed there—from hatching to 35-40 days post-hatch (dph). We obtained 20-45 recordings of sounds in the room on random days and times of the day. Each recording lasted 10 minutes. Because male zebra finches sing only one song type, we were able to detect each event of singing by each male by listening to the recordings or looking at their spectrograms. We defined an event of singing as a continuous bout of song lasting 3 seconds or less. We chose this threshold because it corresponded to the duration of the shortest bout of singing in a sample of the recordings. The average number of events per recording was our estimate of singing rate. Among the three neighbors, the bird with the song rate closest to that of the father was selected as the neighbor in the key-pressing assay.

For each pupil, the playbacks triggered by key-pressing consisted of one recording of father's song or one recording of neighbor's song. Each recording comprised two consecutive song motifs, or units of song, from the song of the corresponding bird. Motifs consist of a series of distinct sounds, known as syllables, that are generally sung in the same order by a bird.[1] To record the motifs, we separated the male from its female partner for 20 minutes. We then reunited the pair, which prompted the male to sing to the female. The song was recorded using a TASCAM DR-7MKII recorder. The stimulus was high-pass filtered at 800 Hz to eliminate low-frequency noise produced by the recording system. When Logitech Z200 speakers were used, we applied an equalization preset to the stimuli to control for frequency-dependent distortions caused by these speakers.

For both father and neighbor, we tried to select motifs that did not contain calls from the female. It is possible, however, that some of them did. None of the pupils seemed to incorporate a female call into their own song.

Reinforcement schedule

The probability that a key would play its associated song changed throughout each daily session to prevent the preferred song from being played many more times than the other. At the beginning of the session, if the bird pressed only the key associated with its preferred song, the probability of playing that song was 0.75. In other words, out of every four presses, three resulted in playback of the preferred song and one in playback of the other song. The probability was this high at the beginning of each day to strengthen the association between the key and the song. After 12 consecutive presses, the probability went down to 0.67, and to 0.5 after 12 more presses.

When the probability of playing the preferred song was 0.5, the association between the keys and the songs was maintained because under such a scenario the other key was programmed to never play the preferred song. That key played only the song with which it was associated, until that song had been heard that day the same number of times as the preferred song. In this way,

until the preferred song was exhausted, the probability of hearing the associated song for each key was always much higher than hearing the other song.

When a pupil switched keys, the first press after the switch always resulted in playback of the song associated with the newly pressed key, regardless of how many times that song had been played. This rule was introduced to help the bird learn the associations between the keys and the songs. Moreover, if the bird did not have a preference and constantly switched between keys, exposure was naturally balanced by playing the song associated with the key being pressed. If the bird had a preference, then the 30 playbacks of the preferred song were exhausted first. Because of the probabilistic contingencies, by the time the preferred song was exhausted, the other song had been played at least 19 times, and therefore it did not take many presses to end the session. After the end of the session, the keys became silent.

Reversals

For pupils with strong preferences for a particular song, we applied a reversal, in other words, we reversed the associations between the keys and the songs. Reversals were applied only once per pupil and occurred during the night, in between sessions of operant conditioning. To determine whether a bird should undergo a reversal, we visually inspected plots of daily preference. We applied the reversal when the scores remained in a range between 0.95-1 or 0-0.05 for several consecutive days (typically three to six days). We considered that presses made after a reversal do not indicate preference until the pupils have learned the new contingencies of the keys. We predicted that during this learning, the pupil would gradually switch from one key to the other. As a result, the proportion of presses for the key associated with the preferred song would immediately drop after the reversal, but then recover over the next several hours to days. We removed data generated immediately after the reversal and for the duration of this recovery (Fig. S1). The duration was determined upon visual inspection of the scatterplot of proportion of presses versus days after reversal. Data were not removed if we did not see a change in proportion of presses for father's song, which we assumed was explained by learning of the new contingencies happening within a day. Of the seven birds that were subjected to a reversal, three learned the new contingencies within a day. For the other four birds, it took between six and eight days to learn the new contingencies. We planned to remove all data collected from pupils that did not switch keys after the reversal, which would have indicated side bias, but all birds that underwent a reversal did switch.

Acquisition, selection, and analysis of pupil songs

We used the software Sound Analysis Pro (SAP)[2,3] to record the vocalizations of the pupils at an age when song is approaching full crystallization.[4–7] Over a ten-day period from 80 to 90 dph, recordings were automatically made of each pupil's vocalizations using the microphone adjacent to the pupil's cage, inside the sound-attenuated chamber, as described in the main Methods. These recordings were up to ten seconds long and typically contained more than one vocalization. Of these recordings, 30 were randomly selected for further analysis. One rendition of the pupil's song was analyzed per each of the 30 recordings. This number of songs is in line with previous studies of song imitation in zebra finches.[8,9] To choose which song within each recording to analyze, we used the function "sample" in R[10] to randomly select a position in each recording and the song closest to that position was chosen.

SAP was developed to automatically record zebra finch vocalizations, but it does not distinguish songs from other vocalizations such as calls.[3] Thus, the recordings included diverse

types of vocalizations. To ensure that the vocalizations chosen for analysis were in fact songs, spectrograms of the recordings were generated in Audacity v 2.2.2[11] using Hanning windows. The window size used for spectral analysis was 512 samples, with overlap between windows of 50%. The value that we chose for the window size allowed us to clearly distinguish frequency contours in the spectrograms. In addition, the corresponding duration of the spectral window (11.61 ms, at a sampling rate of 44100 kHz) was close, within 2-3 milliseconds, to that used in previous studies of song in zebra finches.[2,8,12] Songs were defined as vocalizations that 1) consisted of a train of sounds each less than one second in duration, separated by intervals of silence less than 250 ms in duration, and 2) consecutive sounds in the train differed in shape, as seen in the spectrogram.[1,2]

We then used Sound Analysis Pro 2011 (SAP2011) to evaluate acoustic similarity between the songs of pupils and the songs of each of their tutors in the key-pressing assay, father and neighbor.[2,3] Each of the 30 pupil songs was compared with the rendition of each song that the pupil heard when key-pressing. We took asymmetric measurements of similarity, which determined the extent to which tutor song was copied into pupil song and not vice versa.[3] To avoid including silent intervals between syllables in the analysis, we used amplitude thresholds to filter out those intervals. To further eliminate noise, we used a bandpass filter of 800-8000 Hz. In our recordings, this bandwidth included most of the energy in song while excluding prominent bands of noise caused by electronic interference from building appliances. Other settings in SAP 2011 were set at default values, which are calibrated for analysis of zebra finch song.[3]

To test whether the pupils learned father's or neighbor's song better, on average, we compared the pupils' similarity scores for father's song with that for neighbor's song. To do so, we used the mgcv package in R to fit a beta regression model to our data.[10,13] In this model, the dependent variable was the difference between similarity scores for father's and for neighbor's song for each exemplar of pupil song. The differences between these scores are fundamentally restricted to the interval [-1,1]. Beta regression can model restricted data only for the interval [0,1]. Therefore, we transformed the differences to fit this interval using the following formula: transformed difference = 0.5 * (difference + 1). The identity of the father, the neighbor, and the pupil were modeled as random variables. In our model, a statistically significant intercept indicated that the difference in similarity scores between pupil-father and pupil-neighbor was significant.

References

1. Zann, R. A. *The Zebra Finch: A Synthesis of Field and Laboratory Studies*. (Oxford University Press Oxford, 1996).
2. Tchernichovski, O., Nottebohm, F., Ho, C. E., Pesaran, B., Mitra, P. P. A procedure for an automated measurement of song similarity. *Anim. Behav.* **59**, 1167–1176 (2000).
3. Tchernichovski, O. Sound Analysis Pro 2011 User manual. http://soundanalysispro.com/manual-1 (accessed on 5 January 2021) (2011).
4. Johnson, F., Soderstrom, K., Whitney, O. Quantifying song bout production during zebra finch sensory-motor learning suggests a sensitive period for vocal practice. *Behav. Brain Res.* **131**, 57–65 (2002).
5. Kollmorgen, S., Hahnloser, R. H. R., Mante, V. Nearest neighbours reveal fast and slow components of motor learning. *Nature* **577**, 526–530 (2020).
6. Johnston, T. D. Developmental explanation and the ontogeny of birdsong: Nature/nurture redux. *BBS* **11**, 617–630 (1988).
7. Tchernichovski, O., Mitra, P. P., Lints, T., Nottebohm, F. Dynamics of the vocal imitation process: How a zebra finch learns its song. *Science* **291**, 2564–2569 (2001).

8. Moore, J. M., Woolley, S. M. N. Emergent tuning for learned vocalizations in auditory cortex. *Nat. Neurosci* **22**, 1469–1476 (2019).
9. Feher, O., Wang, H., Saar, S., Mitra, P. P., Tchernichovski O. De novo establishment of wild-type song culture in the zebra finch. *Nature* **459**, 564–568 (2009).
10. R Core Team. R: A Language and Environment for Statistical Computing. https://www.R-project.org/ (2021).
11. Audacity Team. Audacity. http://audacity.sourceforge.net/ (2018).
12. Mandelblat-Cerf, Y., Fee, M. S. An automated procedure for evaluating song imitation. *PLOS ONE* **9**, e96484 (2014).
13. Wood, S. *Generalized Additive Models: An Introduction with R 2nd ed.* (CRC Press, 2017).
