# Supplementary material for: Song preferences predict the quality of vocal learning in zebra finches
Source: Sci Rep. 2023 Jan 12;13:605. doi: 10.1038/s41598-023-27708-y (PMC9837092; doi:10.1038/s41598-023-27708-y)
Supplement: Supplementary file 2 — Supplementary Information 2. [file 41598_2023_27708_MOESM2_ESM.pdf]

**Song preferences predict the quality of vocal learning in zebra finches**

Carlos Antonio Rodríguez-Saltos, Aditya Bhise, Prasanna Karur, Ramsha Nabihah Khan, Sumin Lee, Gordon Ramsay, and Donna L. Maney

## SUPPLEMENTARY TABLE AND FIGURES

Table S1. Brood, father, and assigned neighbor for operant conditioning.

| Pupil | Brood number | Father | Neighbor |
|---|---|---|---|
| ZF1529 | 1 | red-JL-092 | blue-JL-040 |
| ZF1534 | 2 | red-JL-092 | golden-JL-016 |
| ZF1535 | 3 | golden-JL-070 | ZF1517 |
| ZF1536 | 3 | golden-JL-070 | ZF1517 |
| ZF1538 | 3 | golden-JL-070 | ZF1517 |
| ZF1541 | 4 | blue-JL-040 | ZF1507 |
| ZF1542 | 4 | blue-JL-040 | ZF1507 |
| ZF1543 | 4 | blue-JL-040 | ZF1507 |
| ZF1544 | 5 | ZF1518 | golden-JL-007 |
| ZF1545 | 5 | ZF1518 | golden-JL-007 |
| ZF1546 | 5 | ZF1518 | golden-JL-007 |
| ZF1555 | 6 | ZF1507 | golden-JL-016 |

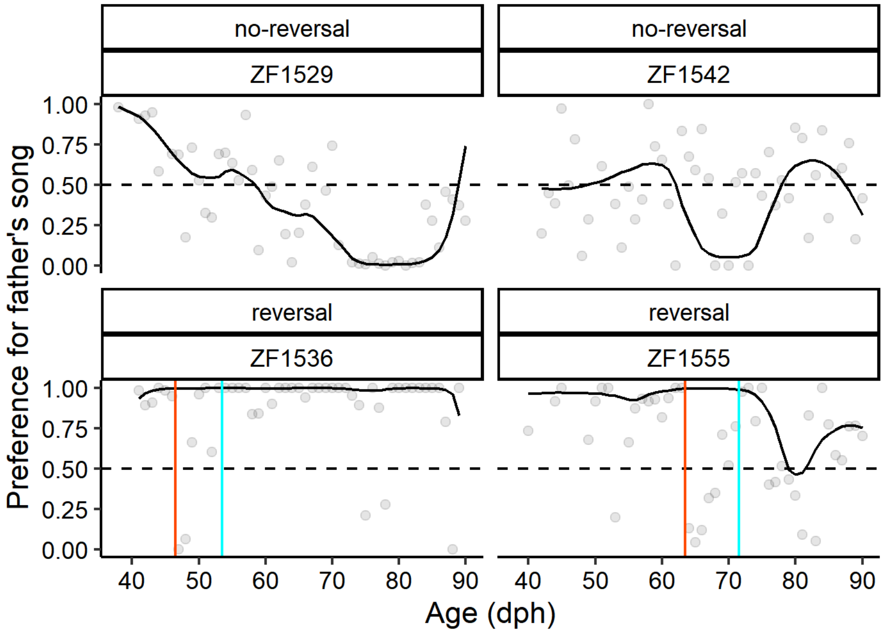


**Figure S1. Examples of trajectories of preference.** The developmental trajectories of preference are shown for four juveniles in this study. The preference for father's song was calculated as the proportion of presses for the key associated with that song. The gray dots in each plot are the daily preference scores for each bird. A smooth trajectory was calculated by applying LOESS on the datapoints. The dashed line indicates a logit preference score of 0.5, at which a bird did not prefer father's or neighbor's song. Scores above 0.5 indicate a preference for father's over neighbor's song. In birds that showed a strong preference for one of the songs (ZF 1536 and ZF 1555), we applied a reversal (red line) to rule out side biases. The blue line indicates the point at which a human observer considered the bird to have finished switching keys after the reversal, meaning that the preference seemed similar to before the reversal. Data between these two vertical lines were not used in LOESS. Despite the original data consisting of proportions, and thus bound to the interval [0-1], LOESS may fit values slightly outside that interval.

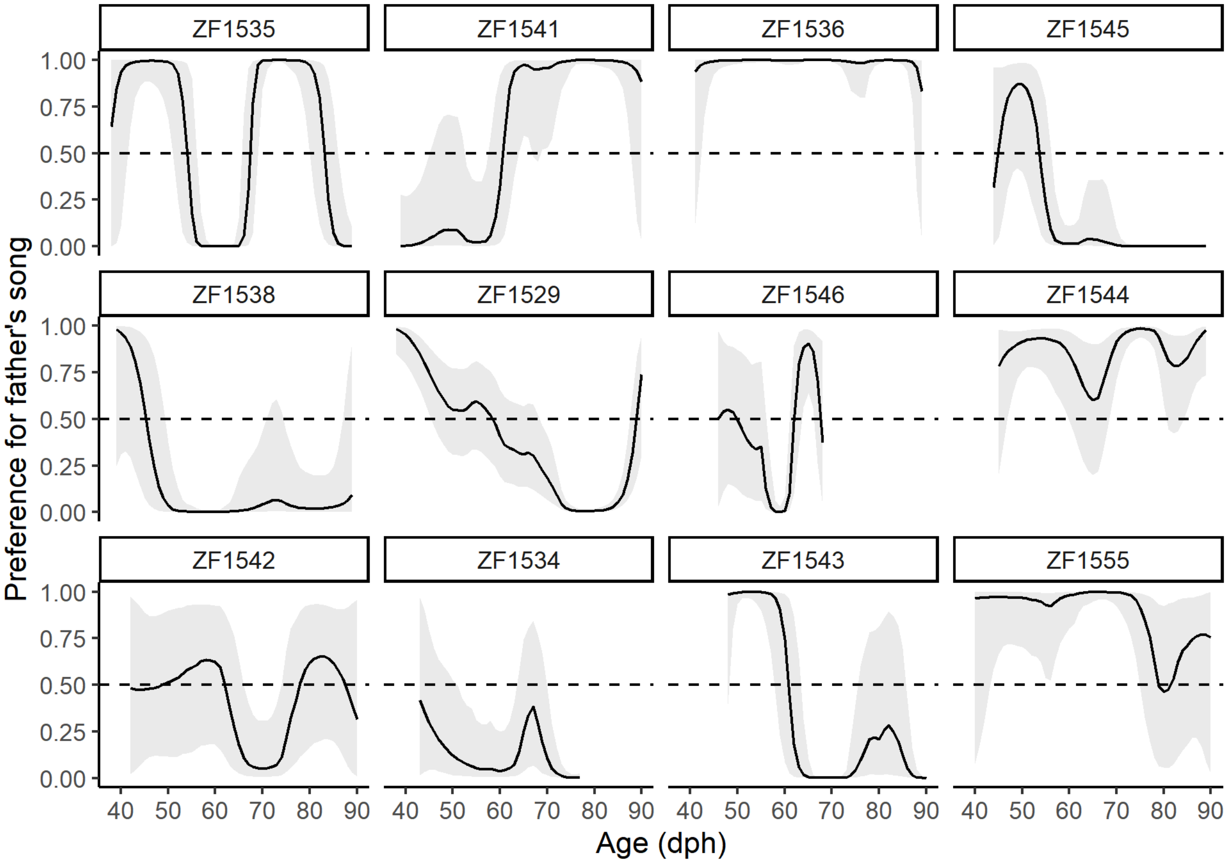


**Figure S2. Individual trajectories of preference for father's song.** The dashed line indicates a logit preference score of 0.5, at which a bird did not prefer father's or neighbor's song. Scores above 0.5 indicate a preference for father's over neighbor's song. For most birds, preference scores for father's song peaked early in development and then decreased. The solid lines are smoothed trajectories fitted to preferences each day (see Fig. S1) using LOESS. The shaded area is the 95% confidence interval of the trajectory. Individual variation in developmental trajectories is to be expected when the trajectories are aligned according to age rather than milestones, which can be reached at different ages in different individuals [1]. Birds are ordered according to the similarity of their songs to their father's song (with most similar at the top), except for the last two birds, for which we could not record their song.

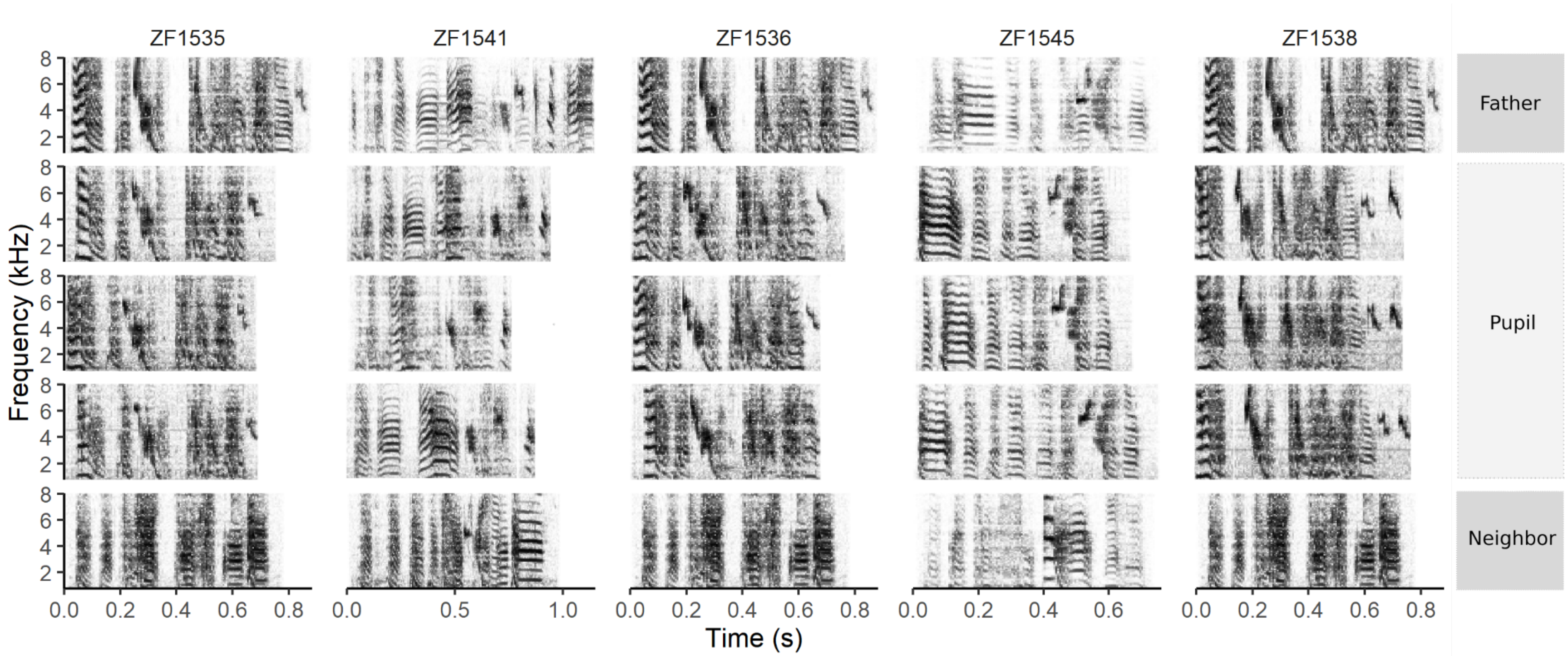


**Figure S3 (Part 1). Comparison of spectrograms of father's, pupil's, and neighbor's songs.** In all cases, the songs of the pupils resembled father's song rather than neighbor's song. Some songs were trimmed to eliminate silence and introductory notes. Syllables in pupils' songs have been reordered for easier visual comparison with father's song. When a pupil repeated a syllable, that repetition is represented here. Pupils are organized in decreasing order of average similarity of their songs to their father's song. Five pupils are shown in this panel and five on the next page.

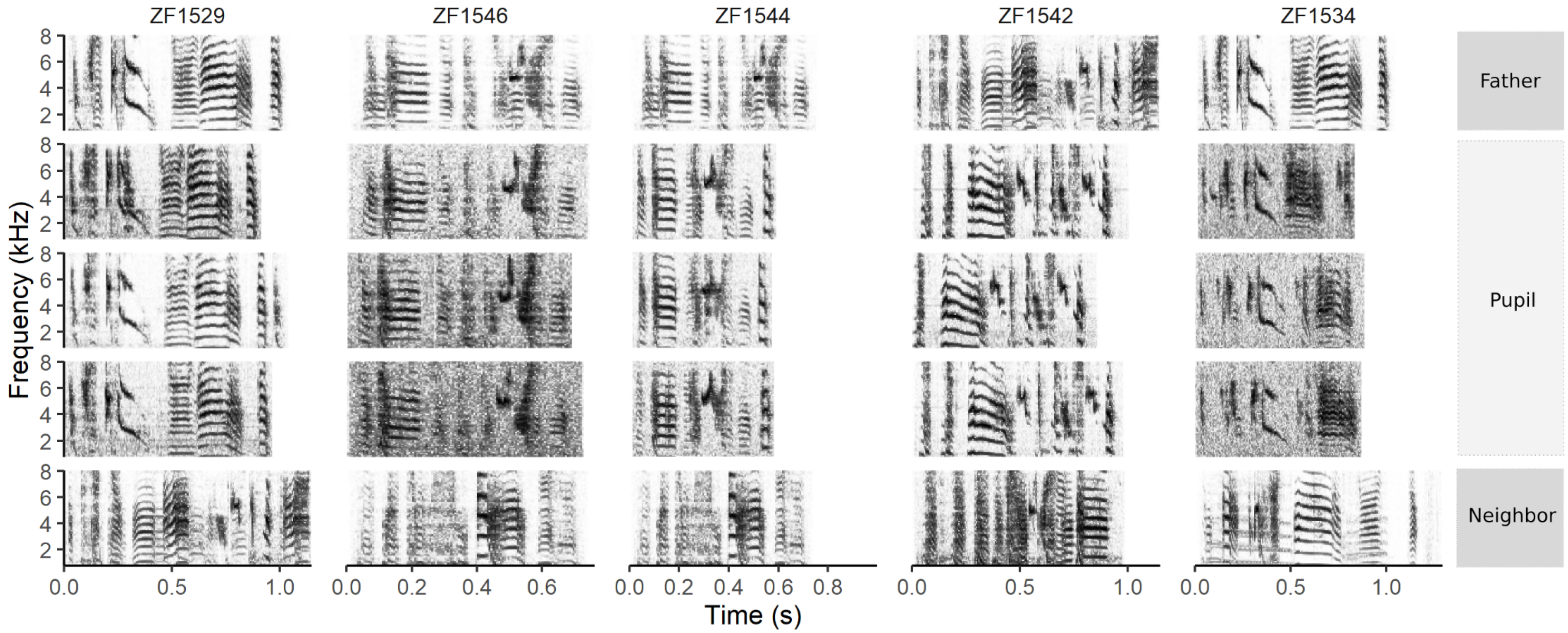


**Figure S3 (continued from previous page).**

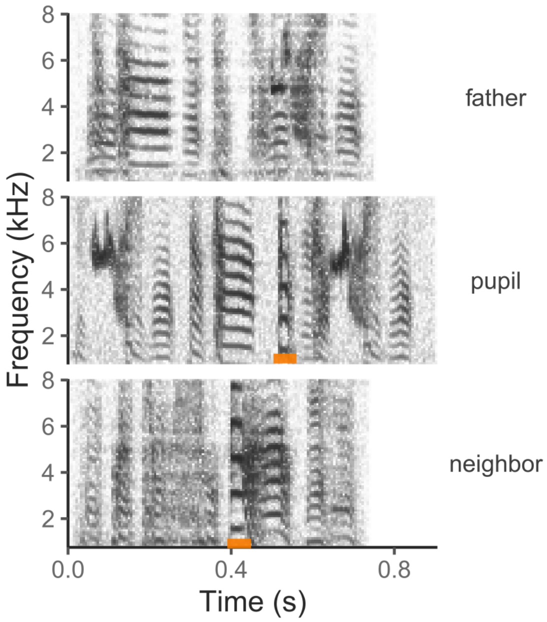


**Figure S4. An element copied from neighbor's song.** In the song of one pupil (ZF1544), we found one element from the song of the corresponding neighbor. The rest of the song, however, resembled father's song. Orange rectangles mark the element in pupil's song and its putative source in the pupil's song.

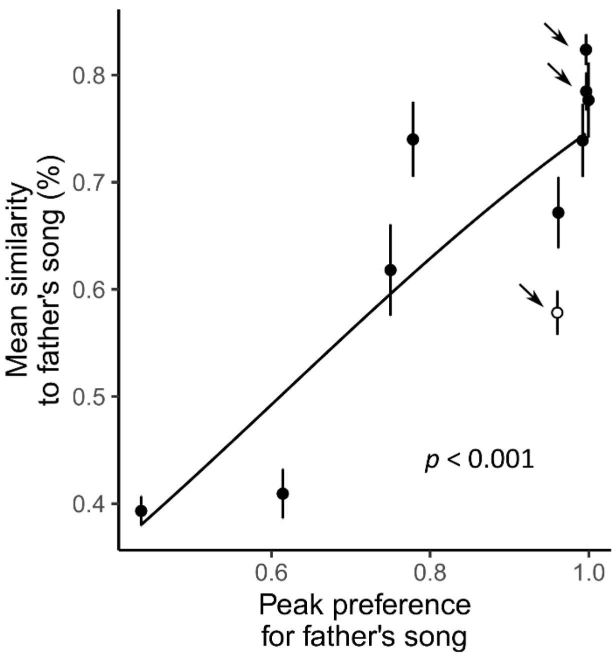


**Fig. S5. Peak preference for father's song, between ages 35 and 90 days post hatch (dph), significantly predicts similarity to father's song.** The strength of the correlation (adjusted R2 = 0.69, p < 0.001, power= 0.92) was no higher than the value estimated when excluding preference after age 70 dph (compare with Fig. 3B). Arrows point to the three birds that reached their peak preference after age 70 dph. The curve is the trend of the beta regression. The white point is an observation that was identified as an influential outlier (Cook's distance > 3 * average Cook's distance). Removing this observation does not affect the significance of the regression. The correlation between peak preference and similarity to neighbor's song was not significant (adjusted R2 = -0.125, p = 0.99, power = 0.05).

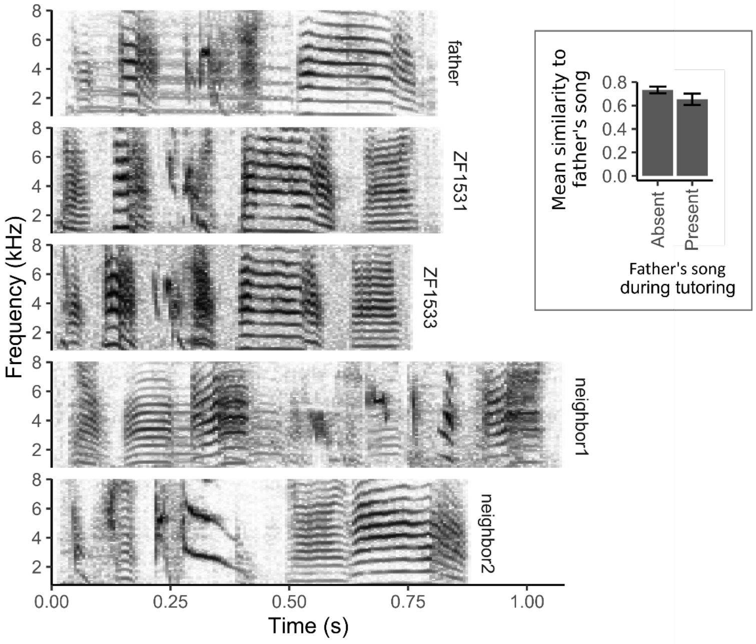


**Fig. S6. Songs of two zebra finches that were not exposed to their father's song during operant conditioning.** From hatching to 39 and 40 days post hatch, the siblings lived in the same cage with their father. Afterwards, during their sensorimotor phase, each sibling was housed in a separate operant conditioning cage and allowed to elicit playback of two adult birds (neighbor 1 and neighbor 2), neither of which were their father. The spectrograms show that despite not having had exposure to their father's song, the siblings imitated the father's song and not any of the neighbors' songs. Barplot: Mean similarity to father's song was not lower for these juveniles (father's song absent during operant tutoring) than for the other birds in this study (father's song present during operant tutoring). Given that birds ZF1531 and ZF1533 came from the same brood, it should not be concluded that absence of father's song during operant tutoring leads to better imitation of that song. Error bars correspond to standard error of the mean.

## Reference for the supplementary table and figures

1. Ramsay JO, Silverman BW. 1997 *Functional Data Analysis*. New York: Springer.
